# Comparison of eating disorders and eating behaviors in adults with and without type 2 diabetes prior to bariatric surgery

**DOI:** 10.1186/s40337-022-00623-9

**Published:** 2022-07-19

**Authors:** Zeinab Shakeri, Farzaneh Mardali, Maedeh Azizabadi Farahani, Mehdi Alemrajabi, Azadeh Mottaghi

**Affiliations:** 1 Department of Nutrition, Science and Research Branch, Islamic Azad University, Tehran, Iran; 2 Department of Nutrition, School of Public Health, Iran University of Medical Sciences, Tehran, Iran; 3 Maragheh University of Medical Sciences, Maragheh, Iran; 4 Iran University of Medical Sciences, Tehran, Iran; 5 Research Center for Prevention of Cardiovascular Diseases, Institute of Endocrinology and Metabolism, University of Medical Sciences, Tehran, Iran

## Abstract

**Background:**

Eating disorders (ED) are group of psychological disorders that significantly impair physical health and psychosocial function. ED consists wide range of morbidity such as loss of eating control, binge eating disorder (BED), night eating syndrome, and bulimia nervosa. Eating behavior is a wide range term that includes food choices, eating patterns, eating problems. In this study, we compared eating disorders and eating behaviors in adults with and without type 2 diabetes prior to bariatric surgery.

**Methods:**

284 participants with class III obesity were included in the single center study. Each case (patients with type 2 diabetes) and control (patients without type 2 diabetes) groups consists 142 patients. Loss of eating control, BED and Bulimia nervosa, Night eating syndrome and eating behaviors and psychosocial factors were screened with standard questionnaires. SPSS version 20 was used for statistical analysis. A P-value of < 0.05 was considered significant.

**Results:**

There was a significant difference between participants with and without type 2 diabetes in case of BED (76.3% vs. 47.3%, P = 0.001). The logistic regression model has shown that participants without type 2 diabetes had lower odds of exhibiting BED (OR = 0.28, 95% CI 0.142–0.552). Among participants without type 2 diabetes, men had 65% high odds of BED (OR = 1.65, 95% CI 1.13–2.53) in compare with women. Participants with and without type 2 diabetes with high school degree (OR = 5.54, 95% CI 2.46–9.45, *P* = 0.0001 and OR = 6.52, 95% CI 3.15–10.56, respectively) and moderate depression level (OR = 2.03, 95% CI 0.98–3.95 and OR = 3.12, 95% CI 2.12–4.56, *P* = 0.0001) had higher odds of BED.

**Conclusion:**

These results probably indicate that people with Class III obesity are more cautious about their diet for blood glucose control if they have type 2 diabetes. Future studies are recommended to follow up these patients after surgery to compare weight loss and blood sugar control in patients with and without type 2 diabetes.

## Plain English summary

Present study aimed to comparison of eating disorders and eating behaviors in adults with and without Type 2 diabetes prior to bariatric surgery. 284 patients with class III obesity were enrolled in the study. Half of them had type 2 diabetes. Several questionnaires were completed by patients to assess the eating disorders and eating behaviors. Results of our study showed that patients without diabetes with class III obesity had lower chance for BED and among them; men were 65% more likely than women to have binge eating disorder. Future studies are recommended to follow up these patients after surgery to compare weight loss and blood sugar control in patients with and without type 2 diabetes.

## Introduction

Eating disorders (ED) are group of psychological disorders that significantly impair physical health and psychosocial function [1]. ED has been reported among bariatric surgery candidates with higher rates of psychological problems, including depression and anxiety disorders and low self-esteem [2]. Disturbed attitude to weight and body shape have key role in the development and maintenance of eating disorders [1]. ED consists wide range of morbidity such as loss of eating control, binge eating disorder (BED), night eating syndrome (NES), and bulimia nervosa [3]. BED and NES have been higher in the patients with class III obesity who candidate for bariatric surgery than non-surgery-seeking patients with class III obesity, although ED may influence weight loss after surgery [4]. People with class III obesity often have a mental problem such as depression and ED. These patients usually have disturbed body image, self-worth, empowerment, and interpersonal relationships [5, 6].

BED is defined as food consumption over a discrete period of time (i.e., 2 h), accompanied by feeling of loss of eating control and subsequent discomfort [7]. BED and obesity are strongly related. Patients with BED usually have other medical conditions such as type 2 diabetes. Several risk factors have been related to the incidence of the mentioned disorder [8]. Niego et al. [9] reported that the rate of pre-surgical BED in patients with class III obesity ranged from 2 to 49% and also the rate of clinical binge eating behaviors ranged from 6 to 14% in the different studies. Most patients who had BED or loss of eating control after surgery were those who have history of pre surgery eating disorders; moreover they experience less weight loss or gain more weight after surgery [9].

Eating behavior is a wide range term that includes food choices, eating patterns, eating problems such as obesity, and eating disorders [10]. Eating disorders and eating behaviors are the most common factors that have significant effects on the outcome of surgery in bariatric surgery candidates [10]. Harris et al [11] reported that the prevalence of BED in patients with type 2 diabetes was much higher than the 2–3.5% prevalence seen in the public population. The most common of eating disorders among patients with type 2 diabetes was binge eating and some studies illustrated that up to 20% of patients with type 2 diabetes suffered from eating disorder.

Only two studies have assessed weight loss after bariatric surgery according to having type 2 diabetes. Individuals with obesity and type 2 diabetes tend to less weight loss than people without type 2 diabetes and have more problems in maintaining the weight they have lost [12, 13]. Therefore, bariatric surgery can be considered as the most effective treatment method for weight loss and maintenance specially for type 2 diabetes treatment [14, 15]. Several large cohort studies have shown that patients with type 2 diabetes who underwent bariatric surgery have achieved 74% more improvement than other treatment methods at 1 year of surgery [16–19] while the prevalence of type 2 diabetes in patients with BED is one in three and 90% of those are in the pre type 2 diabetes stage [20].

In this study, current knowledge will be discussed about comparison of eating disorders and eating behaviors in adults with and without type 2 diabetes prior to bariatric surgery.

## Methods

### Study population

The present study is a case-control, single-center study that has done on patients with class III obesity that referred to Firoozgar hospital and candidate of bariatric surgery. Recruitment of patients was based on their referral to an obesity surgery clinic and their willingness to participate in the study.

This study includes 284 participants in the age range 20–70 years old. Each case (patients with type 2 diabetes) and control (patients without type 2 diabetes) groups consists 142 patients. The patient's unwillingness to continue completing the questionnaire at any time during the completion process led to the patient leaving the project. All the participants were signed informed consent. Patients were asked to answer a questionnaire that contained information about sex, age, marital status, educational level and smoking status. Height and weight were evaluated by trained staff using standard protocols and calculated BMI with confirmed equation.

Loss of eating control, BED and Bulimia nervosa, Night eating syndrome and eating behaviors and psychosocial factors were diagnosed with standard questionnaires and interview by clinical psychologist. In the present study we used the questionnaire that validated in “The Longitudinal Assessment of Bariatric Surgery-2 (LABS-2)” [21].

### Assessment of eating disorders and eating behaviors

#### Loss of eating control

The answer of yes to both questions, “During the past 6 months, have you had times when you eat continuously during the day or parts of the day without planning what and how much you would eat?” and “did you experience a loss of control; that is you felt like you could not control your eating?” indicates loss of eating control.

#### BED and bulimia nervosa

Fifth Edition of the Diagnostic and Statistical Manual of Mental Disorders (DSM-5) was used to detection of BED and bulimia nervosa. For determination of bulimia nervosa, some questions about vomiting, using excessive laxatives, diuretics, or diet pills exercise, or fasting or withholding insulin to control weight after binge eating were asked.

#### Night eating syndrome

Nocturnal eating or hyperplasia at the evening was considered as nigh eating syndrome. For detection of participant with night eating syndrome, we designed 3 questions about eating pattern in the evening and night. If all three questions were answered yes, the syndrome would be diagnosed. Those who had night jobs were excluded from this analysis.

#### Assessment of eating behaviors and psychosocial factors

Eating habits includes breakfast, lunch and dinner eating routinely. Eat when do not feel hungry and Eat with a feeling of fullness were assessed with “Almost every day”, “More than one a week”, “One a week” and “Less than one a week”. “Overall health status” and “overall health status compared to last year” and also “daily activities affected by health status” and “energy and emotions levels” were evaluated using questionnaire.

#### Interpersonal support

Interpersonal support was assessed by 12-item validated questionnaire [22]. A better score demonstrates higher interpersonal support.

#### Depression status

Depression symptoms among the last week were asked using the Beck Depression Inventory V.1 (BDI-1) [23]. The question “I have lost more than 5 pounds” was deleted from questionnaire. Because the majority of participants of present study were advises to do so for better surgery outcomes.

#### IWQOL-lite

Impact of weight on quality of life was measured using 31-item questionnaire in 5 areas including self-esteem; sexual life; public distress; and work [24]. Total score and scores in 5 areas separately were reported. A better score demonstrates higher quality of life.

#### Statistical analysis

Frequencies and percentages are reported for categorical data. Mean and standard deviation are reported for continuous data, which were normally distributed. Pearson's chi-square test for categorical variables and independent sample t-test for continuous variables were used to assess statistical significance of differences in baseline characteristics between participants with type 2 diabetes vs. without type 2 diabetes.

A logistic regression was performed to ascertain the effects of type 2 diabetes on the likelihood that participants have BED. Multivariable logistic regression was used to identify factors that were independently related to having BED in participants with type 2 diabetes vs. without type 2 diabetes. The following variables were considered: sex, age, marital status, education, smoking status, regularity of eating each type of meal per week (breakfast, lunch and dinner), frequency of eating snacks/meals, frequency of eating meals from fast food restaurants, frequency of eating meals from any type of restaurant, counseling in the past year for psychiatric or emotional problems, current medication use for psychiatric or emotional problems, level of depression, ISEL score, BDI score and IWQOL-Lite domain scores. Mean was used to report continuous variables. Using backward elimination, non-significant variables were removed from model. Adjusted odds ratios [OR] and 95% confidence intervals [CI] are reported. A p-value of <0.05 was considered significant.

## Results

The current study includes 284 participants who complete questionnaire. Days per week of regular meals and specific meals eaten are shown in Table 1. The majority of participants have been reported eaten lunch (71%) and dinner (61.9%) regularly in 6–7 days per week, while just about half of participants reported regular breakfast (39%). Highest number of restaurant meals and fast food meals were 1–2 days per week. Moreover, about 33% of participants had lunch at restaurant and ate fast food for dinner (45.2% n = 95). In general, the most of participants reported 1–2 extra meal over than regular meals.

**Table 1:** Days per week of regular meals and specific meals eaten

N (%)									
Days per week	Regular meals eaten			Restaurant meals eaten			Fast food meals eaten		
	Breakfast (%)	Lunch (%)	Dinner (%)	Breakfast (%)	Lunch (%)	Dinner (%)	Breakfast (%)	Lunch (%)	Dinner (%)
0	43 (20.5)	7 (3.3)	15 (7.1)	156 (74.3)	99 (47.1)	97 (46.2)	158 (75.2)	96 (45.7)	75 (35.7)
1–2	49 (23.3)	15 (7.1)	25 (11.9)	29 (13.8)	69 (32.9)	67 (31.9)	35 (16.7)	85 (40.5)	95 (45.2)
3–5	36 (17.1)	39 (18.6)	40 (19.0)	19 (9.0)	34 (16.2)	33 (15.7)	12 (5.7)	22 (10.5)	30 (14.3)
6–7	82 (39.0)	149 (71.0)	130 (61.9)	6 (2.9)	8 (3.8)	13 (6.2)	4 (1.9)	6 (2.9)	9 (4.3)

There was no significant difference between some demographic characteristics or other variables (e.g., sex, marital status, smoking status, ghelion use (ghelion is the persian word for hookah) in participants having or not having type 2 diabetes, Table 2. There was significant difference between education level of participants with and without type 2 diabetes. The most of participants without type 2 diabetes had diploma (40%, n = 57) and bachelor degree was common level of education among participants with type 2 diabetes (32.2%, n = 45). 93.2% (n = 132) of participants with type 2 diabetes were non- smoker and 85.9% (n = 122) of participant without type 2 diabetes did not use ghelion.

**Table 2:** Demographic characteristics of participants according to having or not having type 2 diabetes

	Type 2 diabetes (n = 142)	Non-type 2 diabetes (n = 142)	p-value
Age, years	43.69 ± 10.12	36.28 ± 10.48	0.0001
Age group			
< 30	26 (18.6%)	18 (12.7%)	0.89
30–39	5 (3.4%)	6 (4.7%)	
40–49	5 (3.4%)	7 (5.3%)	
50–59	2 (1.7%)	9 (6.0%)	
> 60	104 (72.9%)	102 (71.3%)	
Sex			
Female	120 (84.7%)	111 (78.0%)	0.08
Male	22 (15.3%)	31 (22.0%)	
BMI	44.2 ± 12.5	41.7 ± 11.1	0.035
Marital status			
Single	15 (10.2%)	41 (28.7%)	0.067
Married	120 (84.7%)	97 (68.7%)	
Widow	7 (5.1%)	4 (2.7%)	
Education level			
Illiterate	7 (5.1%)	3 (2.0%)	0.041
High school	34 (23.7%)	35 (24.7%)	
Diploma	34 (23.7%)	57 (40.0%)	
Associate	17 (11.9%)	8 (6.0%)	
Bachelor	45 (32.2%)	25 (17.3%)	
Master	0 (0.0%)	13 (9.3%)	
Doctoral or higher	5 (3.4%)	1 (0.7%)	
Smoking			
Non-smoker	132 (93.2%)	111 (78.0%)	0.267
Smoker	10 (6.8%)	24 (16.7%)	
Smoker in the past	0 (0.0%)	7 (5.3%)	
Ghelion use			
Yes	15 (10.2%)	20 (14.1%)	0.695
No	127 (89.8%)	122 (85.9%)	

Tables 3 illustrates eating patterns and other factors related to health status of participants according to having or not having type 2 diabetes. There was a significant difference between participants with and without type 2 diabetes in case of BED (76.3% vs. 47.3%, *P*= 0.001).

**Table 3:** Eating patterns and other factors related to health status of participants according to having or not having type 2 diabetes

	Type 2 diabetes (n = 142) (%)	Non-type 2 diabetes (n = 142) (%)	P-value
Loss of eating control			
Yes	108 (76.3)	86 (60.7)	0.08
No	34 (23.7)	56 (39.3)	
Binge eating disorder			
Yes	108 (76.3)	67 (47.3)	0.001
No	34 (23.7)	75 (52.7)	
Bulimia nervosa			
Yes	19 (13.6)	12 (8.0)	0.444
No	123 (86.4)	120 (92.0)	
Night eating syndrome			
Yes	3 (1.7)	9 (6.7)	0.34
No	139 (98.3)	133 (93.3)	
Eating habits			
Breakfast eating routinely (6–7 day/week)			
Yes	58 (40.7)	55 (38.7)	0.67
No	84 (59.3)	87 (61.3)	
Lunch eating routinely (6-7 day/week)			
Yes	94 (66.1)	103 (72.7)	0.523
No	48 (33.9)	39 (27.3)	
Dinner eating routinely (6-7 day/week)			
Yes	70 (49.2)	96 (67.3)	0.023
No	72 (50.8)	46 (32.7)	
Eat when do not feel hungry			
Almost every day	27 (18.6)	53 (37.3)	0.151
More than one a week	19 (13.6)	12 (8.7)	
One a week	48 (33.9)	40 (28.0)	
Less than one a week	48 (33.9)	37 (26.0)	
Eat with a feeling of fullness			
Almost every day	31 (22.0)	55 (38.7)	0.024
More than one a week	21 (13.6)	21 (14.7)	
One a week	43 (28.8)	45 (32.0)	
Less than one a week	47 (32.2)	21 (14.7)	
Overall health status			
Excellent	0 (0.0)	7 (4.7)	0.17
Very good	7 (5.1)	6 (4.0)	
Good	24 (16.9)	47 (33.3)	
Fair	70 (49.2)	58 (40.7)	
Poor	41 (28.8)	24 (17.3)	
Overall health status Compared to last year			
Much better	0 (0.0)	8 (6.0)	0.326
A little better	14 (10.2)	8 (6.0)	
Like last year	65 (45.8)	51 (36.0)	
Slightly worse	34 (23.7)	47 (33.3)	
Much worse	29 (20.3)	28 (18.7)	
Daily activities affected by health status			
Very limited	8 (5.1)	2 (1.3)	0.085
Slightly limited	91 (64.4)	71 (50.0)	
No restrictions	43 (30.5)	69 (48.7)	
Energy and emotions			
Most of time	5 (3.4)	0 (0.0)	
Considerable time of day	19 (13.6)	19 (13.3)	0.554
Sometimes	99 (69.5)	97 (68.7)	
A little bit of time	17 (11.9)	25 (17.3)	
Never	2 (1.7)	1 (0.7)	

There were no significant differences in rates of bulimia nervosa and loss of eating control between participants with and without type 2 diabetes.

None of participants with type 2 diabetes had not excellent overall health status and also overall health status compared to last year was not much better.

Psychosocial factors related to weight of participants according to having or not having type 2 diabetes is shown in Table 4. Participants with the least ISEL scores, had higher risk of eating disorders and it is more common among patients with type 2 diabetes (29.31% vs. 30.45%, *p* = 0.050). Results shown that the most of participants with type 2 diabetes had not hospital admission due to psychosocial problems (98.3%, n = 139) and did not use of medication (64.4%, n = 92) due to psychological problems. 83.1% (n = 118) of participants with type 2 diabetes had not mental health problems prior to bariatric surgery. Among the IWQOL-Lite items, there were significant difference between self-esteem score (22.97 ± 6.42 vs. 20.58 ± 8.34, *p* = 0.050) and work score (10.54 ± 5.97 vs. 13.07 ± 5.28, *p *= 0.003) between participants with or without type 2 diabetes.

**Table 4:** Psychosocial factors related to weight of participants according to having or not having type 2 diabetes

	Type 2 diabetes (n = 142)	Non-type 2 diabetes (n = 142)	P-value
ISEL, score	29.31 ± 3.59	30.45 ± 3.801	0.05
Hospital admission due to psychological problems			
Yes	3 (1.7%)	13 (9.3%)	0.15
No	139 (98.3%)	129 (90.7%)	
Use of medication due to psychological problems			
Yes	50 (35.6%)	45 (31.3%)	
No	92 (64.4%)	97 (68.7%)	0.66
Mental health assessment prior to bariatric surgery			
Yes	24 (16.9%)	30 (21.3%)	0.684
No	118 (83.1%)	112 (78.7%)	
BDI score	17.02 ± 10.744	15.02 ± 10.862	0.26
Depression level			
Minimal	56 (38.5%)	71 (50.0%)	
Mild	22 (15.4%)	25 (20.4%)	0.371
Moderate	42 (30.8%)	22 (16.9%)	
Severe	22 (15.4%)	24 (12.7%)	
IWQOL-Lite			
Total score	93.77 ± 20.97	92.25 ± 26.99	0.7
Physical function score	30.25 ± 8.69	30.83 ± 11.02	0.722
Self-Esteem score	22.97 ± 6.42	20.58 ± 8.34	0.05
Sexual life score	12.00 ± 4.74	10.36 ± 6.65	0.09
Public Distress score	17.66 ± 5.00	17.34 ± 5.14	0.69
Work score	10.54 ± 5.97	13.07 ± 5.28	0.003

*ISEL* Interpersonal support evaluation list, *BDI* Beck depression inventory, *IWQOL-Lite* impact of weight on quality of life-lite

The logistic regression model has shown that participants without type 2 diabetes had lower odds of exhibiting BED (OR = 0.28, 95% CI 0.142–0.552).

Adjusted odds ratios for having BED according to having or not having type 2 diabetes are shown in Table 5. Among participants without type 2 diabetes, men had 65% high odds of BED (OR = 1.65, 95% CI 1.13–2.53) in compare with women.

**Table 5:** Adjusted odds ratio (AOR) and 95% confidence interval (CI) for having BED according to having or not having type 2 diabetes

	Type 2 diabetes (n = 142)			Non-type 2 diabetes (n = 142)		
	AOD	95% CI	P-value	AOD	95% CI	P-value
Gender (ref = women)	1.04	0.85–1.62	–	1.65	1.13–2.53	–
Education (ref = illiterate)			0.0001			0.0001
High school	5.54	2.46–9.45		6.52	3.15–10.56	
Diploma	3.18	1.68–7.25		5.43	2.59–10.12	
Associate	2.05	0.92–3.15		3.19	2.03–6.56	
Bachelor	2.45	1.24–4.26		2.95	1.65–4.62	
Master	1.65	1.15–2.36		1.85	1.19–3.03	
Doctoral or higher	1.02	0.75–1.53		1.68	1.31–2.95	
Depression level (ref = minimal)	1.56					0.0001
Mild		1.03–3.42	0.02	1.86	1.05–2.03	
Moderate	2.03	0.98–3.95		3.12	2.12–4.56	
Severe	1.68	0.85–3.26		2.42	1.53–3.33	

Participants with and without type 2 diabetes with high school degree (OR = 5.54, 95% CI 2.46–9.45, *P* = 0.0001 and OR = 6.52, 95% CI 3.15–10.56, respectively) and moderate depression level (OR = 2.03, 95% CI 0.98–3.95 and OR = 3.12, 95% CI 2.12–4.56, *P *= 0.0001) had higher odds of BED.

## Discussion

In the current study, eating disorders and eating behaviors were compared in participants with and without type 2 diabetes prior to bariatric surgery. This cross-sectional study showed positive association between some eating patterns and other factors related to health status of participants with or without type 2 diabetes prior to bariatric surgery. Furthermore, dinner eating routinely (6–7/weeks) and eat with a feeling of fullness were more common in participants without type 2 diabetes. Results showed that participants without type 2 diabetes had lower odds of exhibiting BED. Although among participants without type 2 diabetes, men had 65% high odds of BED in compare with women. Moreover, all participants with high school degree and moderate depression level had higher odds of BED. We also assessed ISEL score and IWQOL-Lite in participants with type 2 diabetes and without type 2 diabetes prior to bariatric surgery. Participants with type 2 diabetes had the lowest ISEL score, self-esteem score and work score than participants without type 2 diabetes.

Ana Raevuori et al. in context of cohort study with 2342 participants have investigated the prevalence of BED in participants with type 2 diabetes. Their results showed that odds of BED in participants with type 2 diabetes were 15.2% higher than participants without type 2 diabetes [25]. However, several studies have shown no significant association between BED and the risk of type 2 diabetes [26–28]. Krishnamurthy et al [26] reported that there was no significant association between BED, age and type 2 diabetes among 512 participants during a mean 2 years of follow-up. Our results demonstrate that participants without type 2 diabetes had lower odds of exhibiting BED. This controversy may be due to that, it was a hospital-based study and may not be show of the prevalence at a population level or excluded participants with history of psychiatric comorbidities, or differences in the assessment instruments of risk of ED and the differences in eating behaviors associated with type 2 diabetes [26–28]. In our study, high risk of BED was observed in men without type 2 diabetes, while J. Kenardy et al. [29] observed that BED is more common in women with type 2 diabetes. Patients with BED often present high comorbidity such as type 2 diabetes and other psychological problems. Several risk factors have been related to the development and maintenance of BED. Moreover, despite some effective treatments for BED, only few patients have ever received special treatment [8].

Previous studies assessed the association between higher BMI and BED in participants with or without type 2 diabetes [30, 31]. While Mannucci et al. [28] reported BED observed less in participants with type 2 diabetes and obesity. Several studies have shown that dinner eating routinely, eat with a feeling of fullness and eating fast food are positively associated with BED in participants with or without type 2 diabetes prior to bariatric surgery [32, 33]. Depression and BED has been significantly associated with type 2 diabetes and these findings indicate that depression have a role in type 2 diabetes development [34].

BED and NES that are common in obesity may affect the management as well as long term outcomes of T2DM. BED through the consumption of huge amounts of calories over a short period of time and other behaviors such as prolonged fasting may affect glycaemic control [35].

Previous studies found that BED has a negative effect on quality of life [36, 37]. While, in the cross-sectional study by Ana et al. [38], that conducted on 96 adults, there was no significant association between BED and quality of life, which may due to the limitations imposed by sample size.

In our study, risk of BED and type 2 diabetes was evaluated in participants with class III obesity. Results showed that the risk of BED in participants without type 2 diabetes was 72% more than others.

Our findings confirm the results of the Jesús’s study, which reported a higher risk of developing BED in men without type 2 diabetes. Furthermore in this investigation, association between eating behavior disorder and age were observed in participants with type 2 diabetes [39]. In contrast with our result, data from the García-Mayor RV’s study which is a cohort study on 517 participants showed that a positive association between BED and type 2 diabetes. This association was significantly higher in male [40]. In study on 2,580 participants, there was significant association between depression and type 2 diabetes [34]. Besides, a result of another study [32] presented that participants without type 2 diabetes with college education or higher, has been higher odds of BED prior to bariatric surgery. One of the reasons that why patients with type 2 diabetes and class III obesity are more likely to develop BED than patients with class III obesity and without type 2 diabetes is that patients with type 2 diabetes are obsessed about what they eat than other people due to high blood sugar, and this issue can lead to develop some disorder related with eating.

Lack of treatment for eating disorders in patients with type 2 diabetes can affect their glycemic control and weight loss after surgery. By recognizing these disorders before surgery and helping to treat them, patients can benefit more from surgery.

Our study has some limitations. First, because of the cross-sectional study design, causal relationships cannot be established. Second, disproportionate number of females to males causes lack of generalizability of this research to the general population of patients with class III obesity. Third, this study is a single-centered and the sample size was modest.

However, the current study also has some strength. In our study we used validated standard methods for assessment, which provided an accurate estimation of BED and eating behavior. Also, we used validated health status tools such as ISEL, IWQOL-Lite, BDI questionnaires to estimate all the mental and psychological problems of participants.

## Conclusion

The result of present study shows that odds of BED was lower in patients without type 2 diabetes with class III obesity, moreover among this category, men were 65% more likely than women to have BED. Patients with class III obesity who had high school degree and moderate depression level had more odds for BED. These results probably indicate that people with class III obesity are more cautious about their diet for blood glucose control if they have type 2 diabetes, and this issue may lead to eating disorders. It is suggested that future studies follow up these patients after surgery to compare weight loss and blood sugar control in patients with and without type 2 diabetes.

## Data Availability

Not applicable.
